# Genomic epidemiology study of *Klebsiella pneumoniae* causing bloodstream infections in China

**DOI:** 10.1002/ctm2.624

**Published:** 2021-11-08

**Authors:** Xinmiao Jia, Cuidan Li, Fei Chen, Xue Li, Peiyao Jia, Ying Zhu, Tianshu Sun, Fupin Hu, Xiaofeng Jiang, Yunsong Yu, Bijie Hu, Qing Yang, Mei Kang, Hongjie Liang, Kang Liao, Longhua Hu, Li Gu, Yan Jin, Qiong Duan, Shufang Zhang, Ziyong Sun, Wenxiang Huang, Hong He, Haifeng Shao, Bin Shan, Chao Zhuo, Ping Ji, Rui Zheng, Gang Li, Yingchun Xu, Qiwen Yang

**Affiliations:** ^1^ Department of Clinical Laboratory, State Key Laboratory of Complex Severe and Rare Diseases, Peking Union Medical College Hospital Chinese Academy of Medical Sciences and Peking Union Medical College Beijing China; ^2^ Medical Research Center, State Key Laboratory of Complex Severe and Rare Diseases, Peking Union Medical College Hospital Chinese Academy of Medical Sciences and Peking Union Medical College Beijing China; ^3^ CAS Key Laboratory of Genome Sciences & Information, Beijing Institute of Genomics Chinese Academy of Sciences, China National Center for Bioinformation Beijing China; ^4^ Department of Clinical Laboratory, Beijing Anzhen Hospital Capital Medical University Beijing China; ^5^ Graduate School, Peking Union Medical College Chinese Academy of Medical Sciences Beijing China; ^6^ Institute of Antibiotics, Fudan University Huashan Hospital Shanghai China; ^7^ Department of Laboratory Medicine The Fourth Affiliated Hospital of Harbin Medical University Harbin China; ^8^ Department of Infectious Diseases, Sir Run Run Shaw Hospital Affiliated with the Zhejiang University School of Medicine Hangzhou China; ^9^ Department of Infectious Diseases Fudan University Zhongshan Hospital Shanghai China; ^10^ Department of Laboratory Medicine The First Affiliated Hospital of Zhejiang University Hangzhou China; ^11^ Department of Laboratory Medicine West China Hospital of Sichuan University Chengdu China; ^12^ Department of Laboratory Medicine The First Affiliated Hospital of Guangxi Medical University Guilin China; ^13^ Department of Laboratory Medicine The First Affiliated Hospital of Sun Yat‐Sen University Guangzhou China; ^14^ Department of Laboratory Medicine The Second Affiliated Hospital of Nanchang University Nanchang China; ^15^ Department of Infectious Diseases Beijing Chao‐yang Hospital Beijing China; ^16^ Department of Laboratory Medicine Shandong Provincial Hospital Affiliated with Shandong University Jinan China; ^17^ Department of Laboratory Medicine Jilin Province People's Hospital Changchun China; ^18^ Department of Laboratory Medicine Haikou People's Hospital Haikou China; ^19^ Department of Laboratory Medicine, Tongji Hospital, Tongji Medical College Huazhong University of Science and Technology Wuhan China; ^20^ Department of Infectious Diseases The First Affiliated Hospital of Chongqing Medical University Chongqing China; ^21^ Department of Infectious Diseases The Affiliated Hospital of Qingdao University Qingdao China; ^22^ Department of Infectious Diseases General Hospital of Eastern Theater Command Nanjing China; ^23^ Department of Laboratory Medicine The First Affiliated Hospital of Kunming Medical University Kunming China; ^24^ State Key Laboratory of Respiratory Disease The First Affiliated Hospital of Guangzhou Medical University Guangzhou China; ^25^ Department of Laboratory Medicine The First Affiliated Hospital of Xinjiang Medical University Wulumuqi China; ^26^ Department of Laboratory Medicine The First People's Hospital of Yunnan province Kunming China; ^27^ Department of Laboratory Medicine General Hospital of Ningxia Medical University Yinchuan China

Dear Editor,

1

*Klebsiella pneumoniae* (*K. pneumoniae*, Kpn) bloodstream infection (BSI) has a considerable prevalence and high mortality worldwide.^1^, ^2^, ^3^ The emergence of carbapenem‐resistant BSI‐Kpns, especially those with hypervirulence, poses a challenge for BSI‐Kpn control worldwide.^4^, ^5^, ^6^ We conducted a large‐scale multicenter epidemiological study and in‐depth genomic analysis of BSI‐Kpns in China, describing a complete molecular epidemiological picture (clinical features, sequence types (STs)/serotypes, antimicrobial resistance/hypervirulence, phenotype/genotype) of BSI‐Kpns. We also revealed the correlations between clinical characteristics and the genotypes of BSI‐Kpns.

A total of 239 Kpns were identified by screening 1219 Gram‐negative bacteria causing BSI from 24 representative hospitals in different regions of China in 2018 (Table 1 and Figure 1; Table S1 and Figure S1). A total of 67.36% (161/239) infections were hospital‐onset (HO), and the others were community‐onset (CO). A total of 66.11% (158/239) patients were males, and middle‐aged (41–65 years, 118/239, 49.37%) and aged (>65 years, 81/239, 33.89%) patients accounted for a significantly higher percentage (*p*‐value < 0.0001) (Table 1; Table S2). We further sequenced the whole genomes of 239 BSI‐Kpns using Illumina Technology (Tables S3 and S4). ST analysis indicated that these strains covered 78 different STs, including seven new STs (Table S5). The most common STs were ST11, ST23 and ST65, together accounting for 41% (98/239) of BSI‐Kpns (Table 1). Sixty‐six (84.6%) STs were found in ≤ three BSI‐Kpns each. Serotypes of capsular (K) and lipopolysaccharide (O) antigens were also predicted (Table 1). We detected 50 different *K*‐loci, with K64 (53/239, 22.18%) predominating, followed by K1 (27/239, 11.30%), K2 (27/239, 11.30%), and K47 (11/239, 4.60%). Twelve O‐loci were detected, O1 and O2 were the most common, together accounting for 79.5% (190/239) of BSI‐Kpns (Table 1). The distribution of strains in different regions showed different characteristics: the majority of strains in East China are ST11/K64/O2v1, while the percentage of ST65/K2/O1v2 strains ranks first in North China and ST23/K1/O1v2 strains account for the most in Northeast China, respectively (Figure 1; Figures S2 and S3). Importantly, most ST11 strains were K64/O2v1 (49/65, 75.38%) and K47/OL101(14/65, 21.54%), all ST23 strains were K1/O1v2 and all ST65 strains were K2/O1v2 (Figure 1C,D; Figure S3). Moreover, strains of the same ST clustered in the same evolutionary branch and strains of different serotypes clustered in different sub‐branches inside of the same ST (Figure 1D).

**TABLE 1:** Demographic information and characteristics of the BSI *K. pneumoniae* isolates

	HO	CO	Total
**All**	161 (67.36%)	78 (32.64%)	239 (100%)
**Sex**			
Male	101 (62.73%)	57 (73.08%)	158 (66.11%)
Female	60 (37.27%)	21 (26.92%)	81 (33.89%)
**Age**			
Children (0–6 years)	14 (8.70%)	1 (1.28%)	15 (6.28%)
Early youth (7–17 years)	1 (.62%)	0 (.00%)	1 (.42%)
Youth (18–40 years)	15 (9.32%)	9 (11.54%)	24 (10.04%)
Middle (41–65 years)	74 (45.96%)	44 (56.41%)	118 (49.37%)
Aged (>65 years)	57 (35.40%)	24 (30.77%)	81 (33.89%)
**Districts**			
East China	74 (45.96%)	35 (44.87%)	109 (45.61%)
South China	27 (16.77%)	18 (23.08%)	45 (18.83%)
Southwest China	25 (15.53%)	6 (7.69%)	31 (12.97%)
Northeast China	23 (14.29%)	6 (7.69%)	29 (12.13%)
North China	7 (4.35%)	6 (7.69%)	13 (5.44%)
Central China	5 (3.11%)	4 (5.13%)	9 (3.77%)
Northwest China	0 (.00%)	3 (3.85%)	3 (1.26%)
**Sequence type**			
ST11	56 (34.78%)	9 (11.54%)	65 (27.20%)
ST23	10 (6.21%)	11 (14.10%)	21 (8.79%)
ST65	9 (5.59%)	3 (3.85%)	12 (5.02%)
ST15	7 (4.35%)	3 (3.85%)	10 (4.18%)
ST29	6 (3.73%)	3 (3.85%)	9 (3.77%)
Other STs	73 (45.34%)	49 (62.82%)	122 (51.05%)
**Capsular (*K*) serotype**			
K64	44 (27.33%)	9 (11.54%)	53 (22.18%)
K1	12 (7.45%)	15 (19.23%)	27 (11.30%)
K2	18 (11.18%)	9 (11.54%)	27 (11.30%)
K47	12 (7.45%)	2 (2.56%)	14 (5.86%)
K54	7 (4.35%)	4 (5.13%)	11 (4.60%)
Other Ks	68 (42.24%)	39 (50.00%)	107 (44.77%)
**Lipopolysaccharide (*O*) serotype**			
O1v2	45 (27.95%)	31 (39.74%)	76 (31.80%)
O2v1	44 (27.33%)	9 (11.54%)	53 (22.18%)
O1v1	25 (15.53%)	18 (23.08%)	43 (17.99%)
O2v2	12 (7.45%)	6 (7.69%)	18 (7.53%)
OL101	13 (8.07%)	4 (5.13%)	17 (7.11%)
Other Os	22 (13.66%)	10 (12.82%)	32 (13.39%)

**FIGURE 1:**
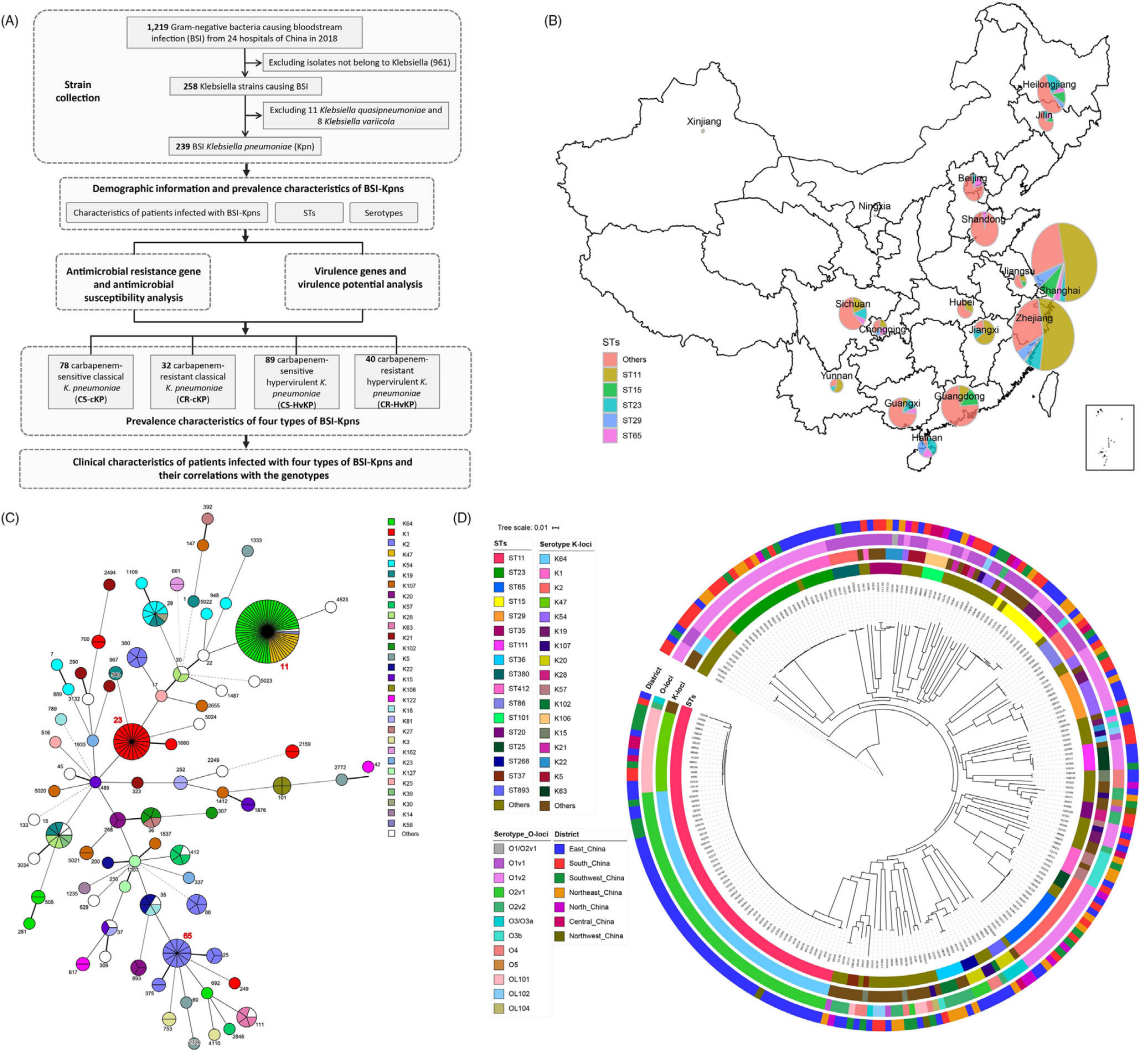
Study design, geographic distribution and phylogenetic analysis of bloodstream infection (BSI) *K. pneumoniae* isolates included in this study. (A) Study design and working flow for this systematic epidemiological study. (B) Collection sites for all *K. pneumoniae* isolates coloured by chromosomal multilocus sequence types (STs) as in the pie chart. (C) Minimum spanning tree of STs as determined by multilocus sequence typing coloured by *K*‐loci. The size of the nodes reflects the number of isolates contained within that particular clade. (D) Phylogenetic tree of core gene SNPs. STs, *K*‐loci, O‐types, and different locations are marked with different colours from inside to outside

Acquired antimicrobial resistance (AMR) gene analysis showed that all 239 strains acquired AMR genes conferring resistance to more than three drug classes (Table S6; Figures S4 and S5). The prevalence of aminoglycosides/chloramphenicol/quinolone/sulph‐onamides/tetracycline/trimethoprim/beta‐lactamase resistance genes all exceeded 30%, with beta‐lactamase predominating (100%) (Table S7; Figure S4). Among beta‐lactamase, the prevalence of carbapenemase genes was 29.71% (71/239) (Table S8). All isolates with carbapenemase were predicted to be resistant to a median of eight drug classes (Figure S6). The most common carbapenemase was *bla*_KPC‐2_, varying widely between different STs/serotypes (93.85% [61/65] ST11, 100% [14/14] K47, 86.79% [46/53] K64 strains and 0% of ST23/ST65/K1/K2 strains) (Table S8, Figure S4C). The prevalence of extended‐spectrum beta‐lactamase genes was 63.6% (152/239), with the most common type being *bla*_SHV_ and *bla*_CTX‐M_ (Table S8). *bla*_CTX‐M_ with the main subtypes of *bla*_CTX‐M‐65_ (56.18%, 50/89) and *bla*_CTX‐M‐3_ (16.85%, 15/89) varied between STs/serotypes, >73% in ST11/K64/K47 and <5% in ST23/ST65/K1/K2 (Table S8; Figure S4C). AmpC genes were present in only 4.6% (11/239) of strains with gene types *bla*_CMY_ and *bla*_DHA_ (Table S8). Eleven antibiotics were used for the antimicrobial susceptibility testing, and the resistant phenotype was consistent with the genotype (Table S9; Figure S4A).

Virulence determinant analysis (Table S10; Figure S7) showed that aerobactin (119/239, 49.79%)‐ and salmochelin (82/239, 34.31%)‐encoding genes, *peg344* (113/239, 47.28%), *rmpA* (113/239, 47.28%) and *rmpA2* (98/238, 41%), which have been suggested to be the most predictive for hypervirulence,^7^ were detected in >30% BSI‐Kpns. They were more prevalent in ST23/ST65/K1/K2 (100% in ST23/ST65; 96.3% in K1/K2 except for *rmpA2*). ST23‐K1/ST65‐K2 contained almost all types of virulence genes, while ST11‐K64/ST11‐K47 strains had fewer (Table S10; Figure S7A,B). The yersiniabactin locus was present in 60.25% (144/239) of BSI‐Kpns, and its prevalence did not differ significantly among different STs/serotypes. Although allantoinase, colibactin and microcin genes were present in <30% of strains, they were more prevalent in ST23/K1 (*p*‐value < 0.001). *Kvg* genes, carried by 13.81% (33/239) of isolates, were more likely to be enriched in ST65/K2 (*p*‐value < 0.001) (Table S10; Figure S7A,B). Most virulence genes of the same type appeared together in clusters, such as the aerobactin‐encoding genes *iucABCD* (Figure S7C). Virulence potential tested by the *Galleria mellonella* infection model showed that 110 out of 129 strains carrying the predictive hypervirulence genes and showed a high virulence potential. There was no significant difference between carbapenem‐sensitive and carbapenem‐resistant strains carrying the predictive hypervirulence genes in virulence potential (Figure S8).

Through the above genotype analysis, four types of BSI‐Kpns possessing different incidence rates and ST/serotype characteristics were identified: carbapenem‐sensitive classical Kpns (organisms carrying no hypervirulence predictive genes^7^) (CS‐cKPs, 78/239, 32.64%) had 49 STs and 38 serotypes; carbapenem‐resistant classical Kpns (CR‐cKPs, 32/239, 13.39%) were dominated by ST11/K64 and ST11/K47; carbapenem‐sensitive hypervirulent Kpns (CS‐HvKPs, 89/239, 37.24%) covered 29 STs and were dominated by ST23/K1 and ST65/K2; carbapenem‐resistant hypervirulent Kpns (CR‐HvKPs, 40/239, 16.74%) were dominated by ST11/K64 (36/40, 90%), and no ST23‐K1 or ST65‐K2 CR‐HvKPs were discovered (Figure 2, Figure S9, Table S11). Detail clinical data were further collated and compared among these four groups (Figure 3). There was a higher ratio of HvKPs in CO isolates (41/67, 61.2%) (Figure 3A,B), and there were no significant differences in the fever peak or procalcitonin among these four groups (Figure 3C,H). However, the values of C‐reactive protein (CRP), neutrophil percent (NEU) and total white blood cells in CR/CS‐HvKPs, especially CRP, were significantly higher than those in CR/CS‐cKPs (Figure 3E–G). The average CRP in CR‐HvKPs reached 140.92 mg/L. Fever duration, intensive care unit (ICU) admission and final prognosis were significantly related to CR‐c/HvKP (Figure 3D,I,J). Further correlation analysis between antimicrobial‐resistance/virulence genes and clinical symptoms (Figure S10) indicated that antimicrobial resistance genes, especially *bla*_KPC‐2_ and quinolone resistance mutations, had a significantly positive correlation with ICU admission and final prognosis but a significantly negative correlation with the BSI type of CO (Figure S10A,C). In contrast, virulence genes, especially *rmpA*, *rmpA2*, aerobactin (*iucABCD*) and *peg344*, were negatively related to the ratio of ICU admission but had a significantly positive correlation with CRP. Virulence genes microcin (*mceABCDEIJ*) and allantoinase (*allABCDRS*) had a significantly positive correlation with CO but were negatively related to the ratio of ICU admission. The yersiniabactin genes (*ybtAEPQSTUX*) had a significantly positive correlation with fever duration (Figure S10B,D).

**FIGURE 2:**
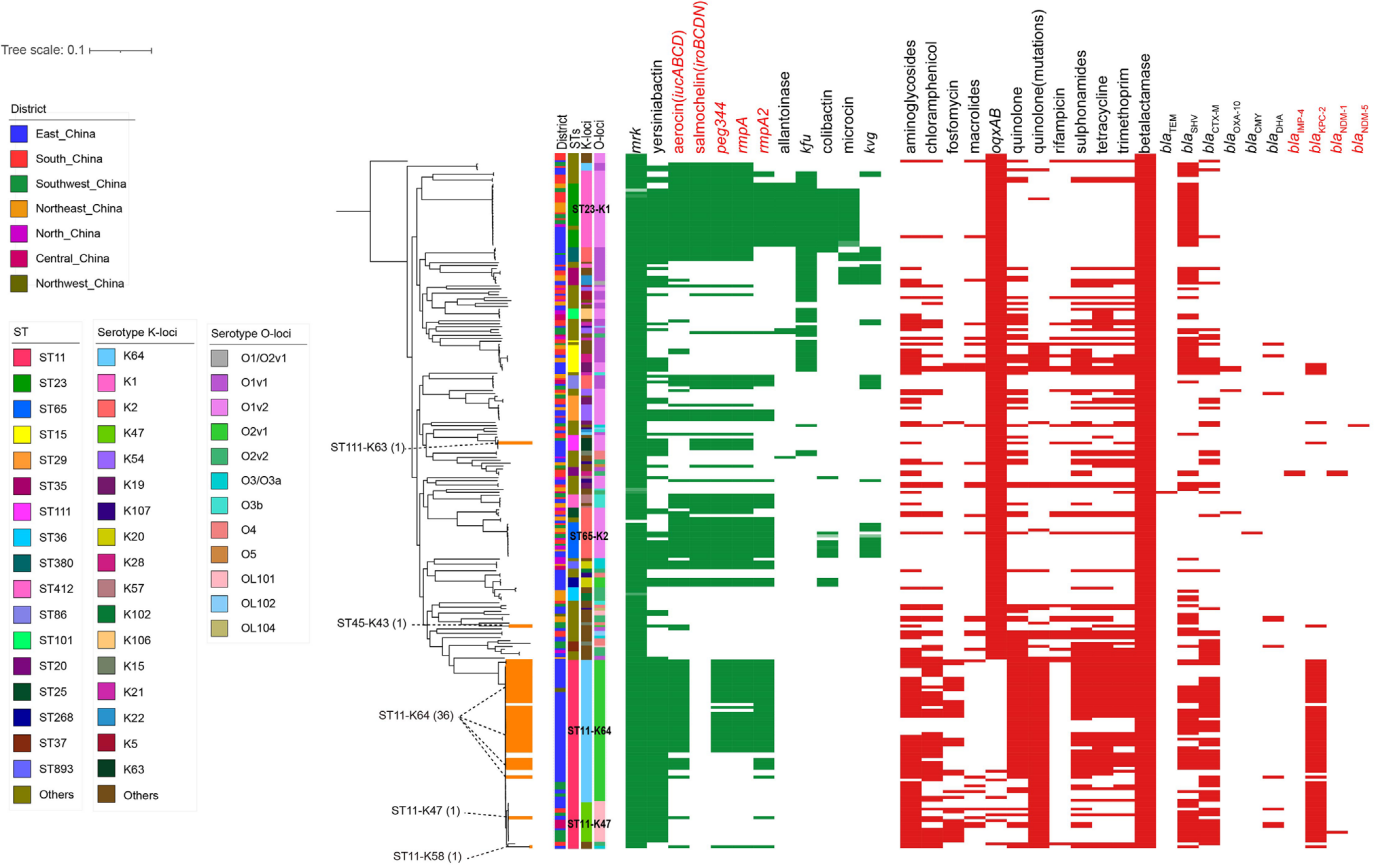
Distribution of virulence genes and antimicrobial resistance genes. Evolutionary relationships, virulence genes and antimicrobial resistance genes are shown from left to right, respectively. The strains not only contained carbapenemases genes (marked in red) but also the hypervirulence genes (marked in red) are highlighted in orange, and their STs and serotypes were also marked

**FIGURE 3:**
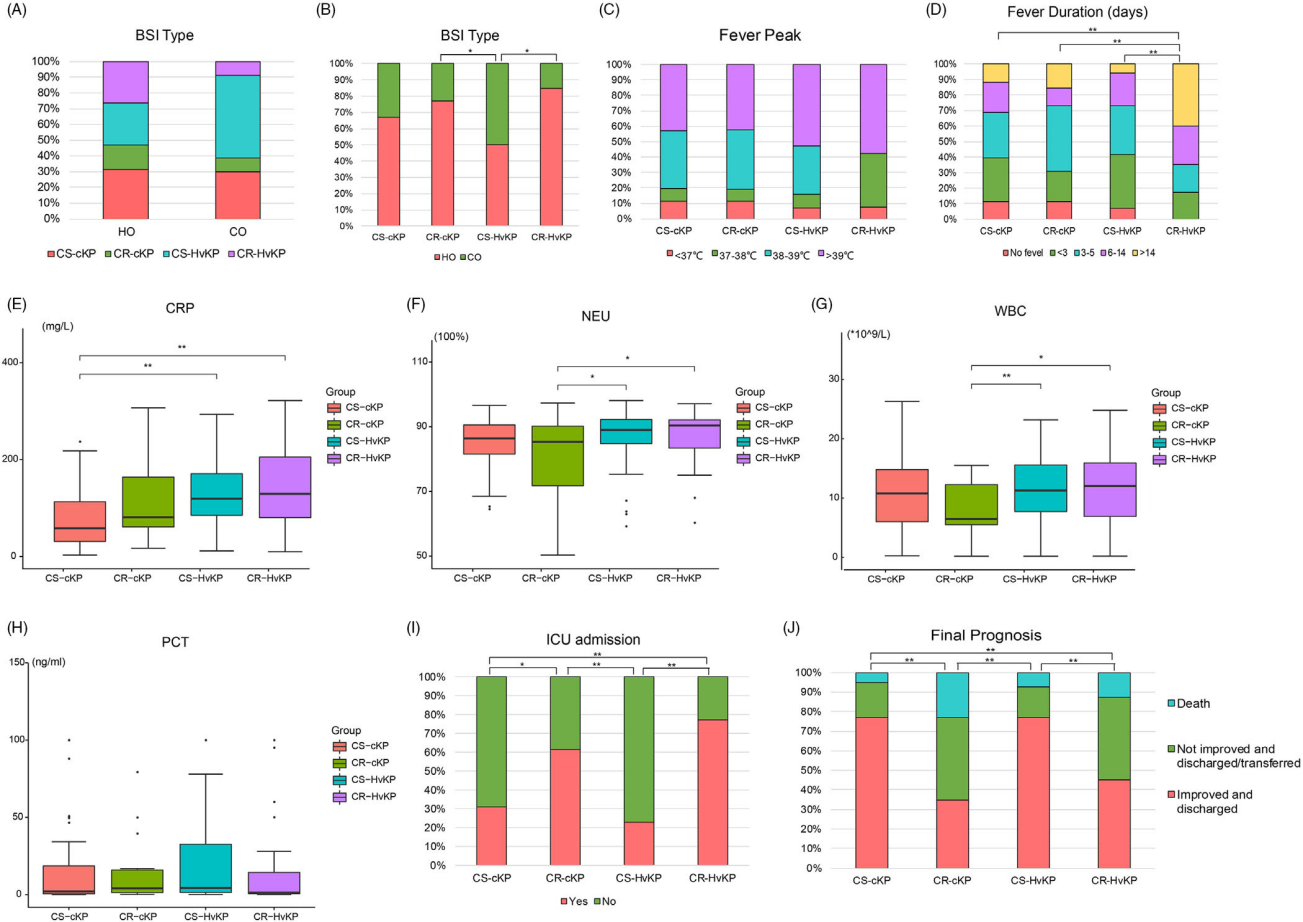
Differences in clinical symptoms and outcomes among patients infected with different types of isolates. (A and B) bloodstream infection (BSI) type, (C) fever peak, (D) fever duration, (E–H) CRP, NEU, PCT, and WBC distribution, (I) ICU admission, and (J) final prognosis. Abbreviations: WBC, total white blood cells; NEU, neutrophil percent; CRP, C‐reactive protein; PCT, procalcitonin; CO, community onset; ICU, intensive care unit. **p‐value < 0.01; *p‐value < 0.05

In conclusion, BSI‐Kpns from China showed a high prevalence of CRKP, HvKP and CR‐HvKP, and different types of strains possessed different STs/serotypes and prevalence features. Patients infected with different types of isolates also showed different clinical symptoms. ST11‐K64 CR‐HvKPs might be a general trend, and other types of CR‐HvKPs have also begun to appear sporadically. Our results provide a complete genomic epidemiological picture and reveal the correlations between clinical characteristics and the genotypes of BSI‐Kpns.

## CONFLICT OF INTEREST

The authors declare no conflict of interest.

## Supporting information

Supporting information

Supporting information
