# Supplementary material for: Genomic epidemiology study of Klebsiella pneumoniae causing bloodstream infections in China
Source: Clin Transl Med. 2021 Nov 8;11(11):e624. doi: 10.1002/ctm2.624 (PMC8574888; doi:10.1002/ctm2.624)
Supplement: Supplementary file 1 — Supporting information [file CTM2-11-e624-s001.docx]

**Supplementary material**

**Genomic epidemiology study of *Klebsiella pneumoniae* causing bloodstream infections in China**

**Authors:** Xinmiao Jia^†1,2^, Cuidan Li^†3^, Fei Chen^†3^, Xue Li^†1,4^, Peiyao Jia^†1,5^, Ying Zhu^†1,5^, Tianshu Sun^1,2^, Fupin Hu^6^, Xiaofeng Jiang^7^, Yunsong Yu^8^, Bijie Hu^9^, Qing Yang^10^, Mei Kang^11^, Hongjie Liang^12^, Kang Liao^13^, Longhua Hu^14^, Li Gu^15^, Yan Jin^16^, Qiong Duan^17^, Shufang Zhang^18^, Ziyong Sun^19^, Wenxiang Huang^20^, Hong He^21^, Haifeng Shao^22^, Bin Shan^23^, Chao Zhuo^24^, Ping Ji^25^, Rui Zheng^26^, Gang Li^27^, Yingchun Xu^1^, Qiwen Yang^*1^

**Affiliations:** ^1^Department of Clinical Laboratory, State Key laboratory of Complex Severe and Rare Diseases, Peking Union Medical College Hospital, Chinese Academy of Medical Sciences and Peking Union Medical College, Beijing, China

^2^Medical Research Center, State Key laboratory of Complex Severe and Rare Diseases, Peking Union Medical College Hospital, Chinese Academy of Medical Sciences and Peking Union Medical College, Beijing, China

^3^CAS Key Laboratory of Genome Sciences & Information, Beijing Institute of Genomics, Chinese Academy of Sciences, Beijing, China

^4^Department of Clinical Laboratory, Beijing Anzhen Hospital, Capital Medical University, Beijing, China

^5^Graduate School, Peking Union Medical College, Chinese Academy of Medical Sciences, Beijing, China

^6^Institute of antibiotics, Fudan University Huashan Hospital, Shanghai, China

^7^Department of Laboratory Medicine, The Fourth Affiliated Hospital of Harbin Medical University, Harbin, China

^8^Department of Infectious Diseases, Sir Run Run Shaw Hospital, affiliated with the Zhejiang University School of Medicine, Hangzhou, China

^9^Department of Infectious Diseases, Fudan University Zhongshan Hospital, Shanghai, China

^10^Department of Laboratory Medicine, The First Affiliated Hospital of Zhejiang University, Hangzhou, China

^11^Department of Laboratory Medicine, West China Hospital of Sichuan University, Chengdu, China

^12^Department of Laboratory Medicine, The first affiliated hospital of Guangxi Medical University, Guilin, China

^13^Department of Laboratory Medicine, The First Affiliated Hospital of Sun Yat-Sen University, Guangzhou, China

^14^Department of Laboratory Medicine, The Second Affiliated Hospital of Nanchang University, Nanchang, China

^15^Department of infectious diseases, Beijing Chao-yang Hospital, Beijing, China

^16^Department of Laboratory Medicine, Shandong Provincial Hospital affiliated with Shandong University, Jinan, China

^17^Department of Laboratory Medicine, Jilin Province People’s Hospital, Changchun, China

^18^Department of Laboratory Medicine, Haikou People’s Hospital, Haikou, China

^19^Department of Laboratory Medicine, Tongji Hospital, Tongji Medical College, Huazhong University of Science and Technology, Wuhan, China

^20^Department of Infectious Diseases, First Affiliated Hospital of Chongqing Medical University, Chongqing, China

^21^Department of Infectious Diseases, The Affiliated Hospital of Qingdao University, Qingdao, China

^22^Department of Infectious Diseases, General Hospital of eastern theater command, Nanjing, China

^23^Department of Laboratory Medicine, the first Affiliated Hospital of Kunming Medical University, Kunming, China

^24^State Key laboratory of respiratory disease, the first affiliated hospital of Guangzhou Medical university, Guangzhou, China

^25^Department of Laboratory Medicine, the first Affiliated Hospital of Xinjiang Medical University, Wulumuqi, China

^26^Department of Laboratory Medicine, the first people’s Hospital of Yunnan province, Kunming, China

^27^Department of Laboratory Medicine, general Hospital of Ningxia medical university, Yinchuan, China

**^†^Contributed equally**

**^*^Correspondence to:**

Prof Qiwen Yang, Peking Union Medical College Hospital, No.1 Shuaifuyuan Wangfujing, Dongcheng District, Beijing, China 100730; Tel: +86 010 69159742; Email: yangqiwen81@vip.163.com

**Key Words:** *Klebsiella pneumoniae*, carbapenem-resistant, hypervirulent, bloodstream infection, ST11-K64

**Supplementary Methods**

**Bacterial strains**

We performed a multicentre epidemiological study to investigate the prevalence and characteristics of hypervirulent and carbapenem-resistant *K. pneumoniae* isolates from patients with bloodstream infections. Strains in this study were from the Study for Monitoring Antimicrobial Resistance Trends (SMART). SMART is an ongoing global surveillance program, which began in 2002 and aims to monitor the in vitro susceptibility to antimicrobials of aerobic and facultative anaerobic Gram-negative bacilli, with a particular focus on those pathogens producing extended-spectrum β-lactamase (ESBL). In China, the SMART collects data from a representative group of Grade-III Class-A hospitals in 7 regions of mainland China (northeast, north, central, east, south, and southwest). Twenty-four hospitals participated in this study, and an average of 50 BSI gram-negative bacterial strains were consecutively collected from each hospital in 2018 according to the protocol (first 50 consecutively-isolated BSI gram-negative bacilli of year 2018 were enrolled in this study from each hospital). Finally, a total of 239 *K. pneumoniae* isolates, including community-onset and hospital-onset strains, were identified by screening 1,219 gram-negative BSI bacterial strains from 24 hospitals in seven geographic regions of China (east, south, southwest, northwest, north, northeast, and central China) (Figure 1A, Table 1, and Table S1). All cases were clinically confirmed and laboratory-confirmed bloodstream infections diagnosed based on the Johns Hopkins ABX Guide (https://www.hopkinsguides.com/hopkins/view/Johns_Hopkins_ABX _Guide/). All organisms were considered clinically significant and were isolated from blood culture specimens. Isolates were sent to the central clinical microbiology laboratory of Peking Union Medical College Hospital (PUMCH) for identification confirmation using MALDI-TOF MS (Vitek MS, BioMérieux, France) and further Average Nucleotide Identity (ANI) analysis based the sequenced reads using pyani (https://pypi.org/project/pyani/). Any duplicate isolate obtained from the same patient was not included in the analysis. The ethics committee of PUMCH approved the human study protocols (ethics number S-K238) and waived the requirement for obtaining informed consent because of the observational design.

In this study, carbapenem-resistant *K. pneumoniae* (CRKP) was defined as strains that were resistant to at least one of the tested carbapenems, including ertapenem, imipenem, and meropenem.^1^ The resistance breakpoints of MIC is 2 ug/ml for ertapenem, 4 ug/ml for imipenem, and 4 ug/ml for meropenem following the CLSI M100-S30 guidelines.^2^ Hypervirulent BSI-Kpns (HvKPs) were defined by the presence of hypervirulence genes *peg344*, *iroB*, *iucA*, *rmpA*, or *rmpA2* according to published studies. ^3^

**Clinical data collection**

Clinical data were collected from a total of 197 enrolled patients, including age, sex, date of admission, date of isolation, BSI type, infection index including fever, C-reactive protein (CRP), procalcitonin (PCT), neutrophil percent (NEU), white blood cell count (WBC), ICU admission, antimicrobial therapy, and clinical outcomes. The hemogram indexes are the results of the first examination after admission within 1-3 days of the occurrence of a bloodstream infection. Observation points for clinical outcomes were 14 days after the onset of infection and the time of discharge/death. Clinical outcomes include “Improved and discharged”, “Not improved and discharged/transferred”, “Death”. Significance tests of CRP, PCT, NEU, and WBC between different groups were conducted using the Kruskal-Wallis test. Significance tests of BSI type, fever, ICU admission, and clinical outcomes between different groups were conducted using the χ2 test. p-value < 0.05 indicates significant differences were found.

We defined community-onset (CO) BSIs as BSIs that occurred in outpatients or in patients within the first 48 h after hospital admission. Otherwise, the infections were defined as hospital-onset BSIs.^4^ The *K. pneumoniae* isolates that caused hospital-onset BSIs were termed hospital-onset (HO) *K. pneumoniae*.

**Antimicrobial susceptibility testing**

Antimicrobial susceptibility testing was conducted by the broth microdilution method as per the Clinical and Laboratory Standards Institute (CLSI) recommendations.^2^ MICs were interpreted following the CLSI M100-S30 guidelines.^2^ *Escherichia coli* ATCC 25922, *Pseudomonas aeruginosa* ATCC 27853, and *K. pneumoniae* ATCC700603 were used as quality controls. Carbapenem-resistant *K. pneumoniae* (CRKP) was defined as strains that were resistant to at least one of the tested carbapenems, including ertapenem, imipenem, and meropenem. The resistance breakpoints of MIC is 2 ug/ml for ertapenem, 4 ug/ml for imipenem, and 4 ug/ml for meropenem following the CLSI M100-S30 guidelines. ^2^

**Virulence testing in *Galleria mellonella* infection model**

We tested the virulence potential of all 239 isolates in *Galleria mellonella* (*G. mellonella*) larvae weighing approximately 300 mg (purchased from Tianjin Huiyude Biotech Company, Tianjin, China). Overnight cultures of strains were washed with saline and further adjusted with phosphate-buffered saline (PBS) to concentrations of 1 × 10^7^ CFU/mL. We infected *G. mellonella* with 1 × 10^5^ CFU (10 μl of bacterial suspension at a concentration of 1 × 10^7^ CFU/mL). Ten *G. mellonella* were inoculated with each isolate. All experiments were performed in triplicate. A known hypervirulent *K. pneumoniae* strain NTUH-K2044 was used as the high-virulence control.^5^ A classic *K. pneumoniae* strain QD110 identified in this study was used as a low-virulence control. ^6^ PBS was used as the blank control in each experiment. Statistical significance of virulence potential in *Galleria mellonella* infection model between any two of the four groups (CS-cKP, CR-cKP, CS-HvKP, and CR-HvKP) were analyzed using paired t test by GraphPad Prism version 8 (GraphPad Software, Inc., USA).

**Genomic DNA extraction, sequencing, assembly, and annotation**

Whole-genome sequencing was conducted on all 239 BSI *K. pneumoniae* isolates. The TIANamp Bacteria Genomic DNA Kit (TiangenBiotechCo. Ltd., Beijing, China) and an Illumina Genome Analyser 2X (Illumina, San Diego, CA, USA) were used for genomic DNA extraction and shotgun sequencing. Adapters and low-quality sequences were trimmed and filtered using Fastx-tookit (http://hannonlab.cshl.edu/fastx_toolkit/), and SPAdes v3.11 was used for the de novo assembly of these reads.^7^ Whole-genome sequencing was performed using the Pacific Biosciences Sequel System (Pacific Biosciences, Menlo Park, CA, USA). De novo assembly of the genome was performed using HGAP within SMRT Link v5.0.0. Gap closing was completed by PBJelly,^8^ and circularization was achieved by manual comparison according to the alignment result of BLAST and removal of regions of overlap. The final genome was further confirmed by remapping Illumina reads using BWA 0.5.9 and Pilon v.1.13.^9^ Gene prediction and the annotation of assembled sequences were performed via Prokka.^10^ Sequencing reads have been deposited in the National Center for Biotechnology Information (NCBI) Sequence Read Archive (SRA) database under PRJNA692086. Information on the sequencing data is shown in Table S3, and the assembly and annotation information are shown in Table S4.

**Sequence types (STs) and serotype analysis**

STs were determined based on multilocus sequence typing (MLST, http://bigsdb.Pasteur.fr/klebsiella/klebsiella.html) and reconfirmed using SRST2 based on the Illumina reads.^11^ For any strain that did not match an existing ST number, we uploaded the sequencing data to the Klebsiella MLST database (https://bigsdb.pasteur.fr/cgi-bin/bigsdb/bigsdb.pl?db=pubmlst_klebsiella_seqdef& page=login) for a new ST number. Serotypes (capsular (K) and O antigen serotypes) were identified based on the whole-genome data using Kaptive (https://github.com/katholt/Kaptive) together with the BLAST results between the known primer sequences and assembled scaffolds.^12^

**Single nucleotide polymorphism (SNP) identification and phylogenetic analysis**

Paired-end reads of *K. pneumoniae* were mapped to the HS11286 genome (NC_016845.1) using BWA 0.5.9. SNPs were analysed using SAMtools 0.1.19^13^ and VarScan^14^ and filtered with at least 20 reads covered and 75% supported. We also filtered out all the SNPs in the repetitive DNA regions identified by RepeatMasker (http://www.repeatmasker.org/) and those in the mobile genetic elements, including insertion sequences and transposons. The phylogenetic tree was constructed using the core SNPs described in all strains using RAxML^15^ with a bootstrap value of 500 and visualized using iTOL (https://itol.embl.de/). Population structure was identified using the R package hierbaps.

**Antimicrobial resistance genes and virulence gene analysis**

Antimicrobial resistance genes were downloaded from the ResFinder 3.0 files in SRST2^11^ and the Comprehensive Antibiotic Resistance Database (CARD). Virulence genes were downloaded from the *K. pneumoniae* BIGSdb (http://bigsdb.Pasteur.fr/klebsiella/klebsiella.html) and Virulence Factor Database (VFDB). The virulence and antimicrobial resistance genes in our sequenced strains were predicted using SRST2 and confirmed by BLAT and kleborate.^16^ The Pearson correlation coefficients for pairwise virulence genes were computed using the corr function and visualized using the pheatmap function in R. Significance tests of virulence gene distribution were conducted using χ2 test.

**Correlation analysis between genotypes and clinical data**

Clinical data on patients were collected from 24 hospitals. Correlations between antimicrobial resistance gene classes and clinical data, correlations between virulence gene classes and clinical data, correlations between antimicrobial resistance genes and clinical data, and correlations between virulence genes and clinical data were all calculated by Spearman correlation analysis using the WGCNA corAndPvalue function and presented by pheatmap.

**Supplementary Figures**

**Figure S1**

**
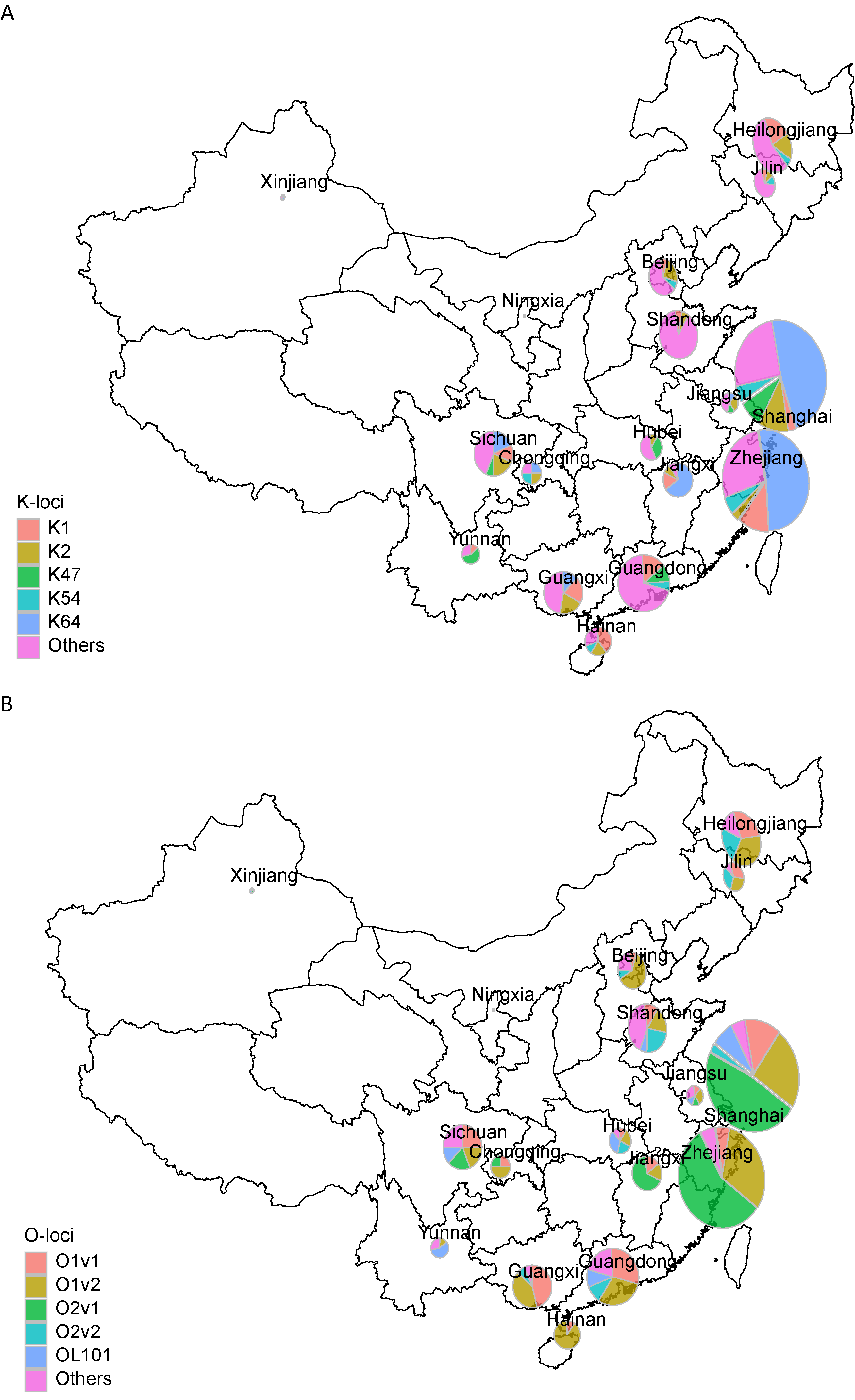
**

**Figure S1 Collection sites of BSI-Kpns from China, 2018.** (A) Collection sites for all *K. pneumoniae* isolates coloured by capsular (K) serotype as in the pie chart. (B) Collection sites for all *K. pneumoniae* isolates coloured by lipopolysaccharide (O) serotype as in the pie chart.

**Figure S2**


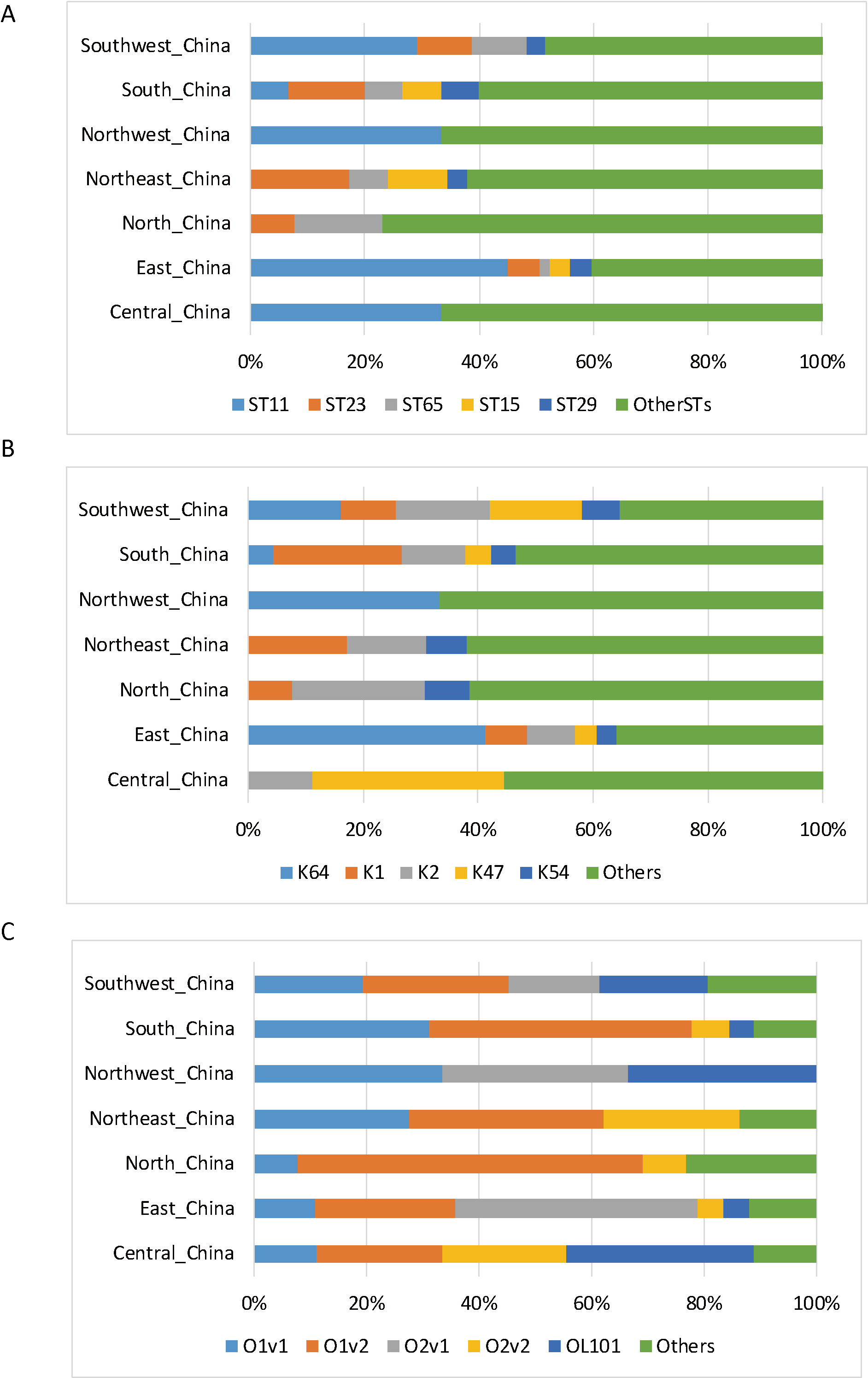


**Figure S2** **Distribution of all *K. pneumoniae* isolates in seven administrative regions.** (A) ST distribution. (B) K-loci distribution. (C) O-type distribution.

**Figure S3**


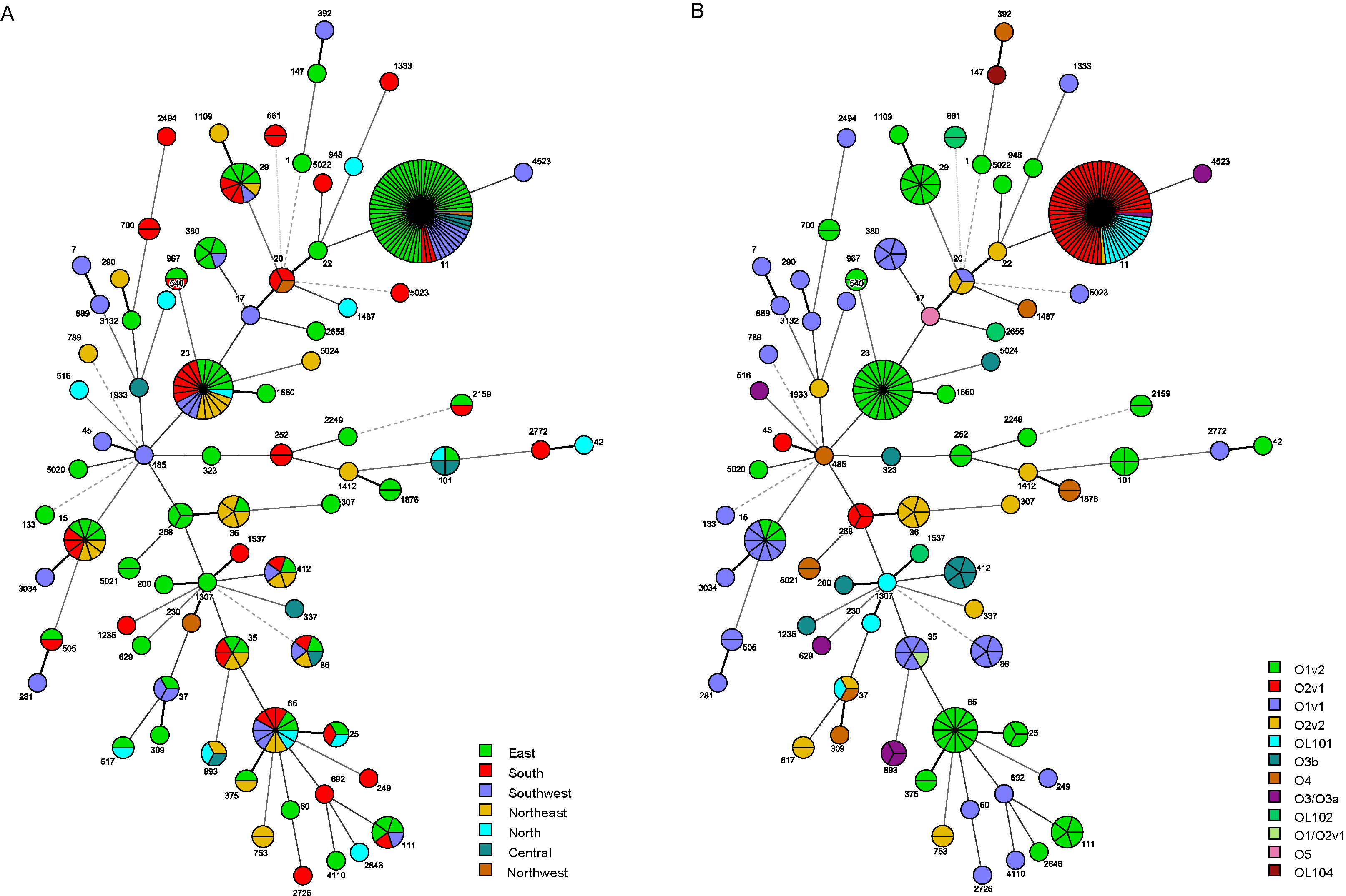


**Figure S3 Phylogenetic analysis of** **BSI-Kpns.** (A) Minimum spanning tree of STs as determined by multilocus sequence typing coloured by seven administrative regions. (B) Minimum spanning tree of STs as determined by multilocus sequence typing coloured by O-type.

**Figure S4**


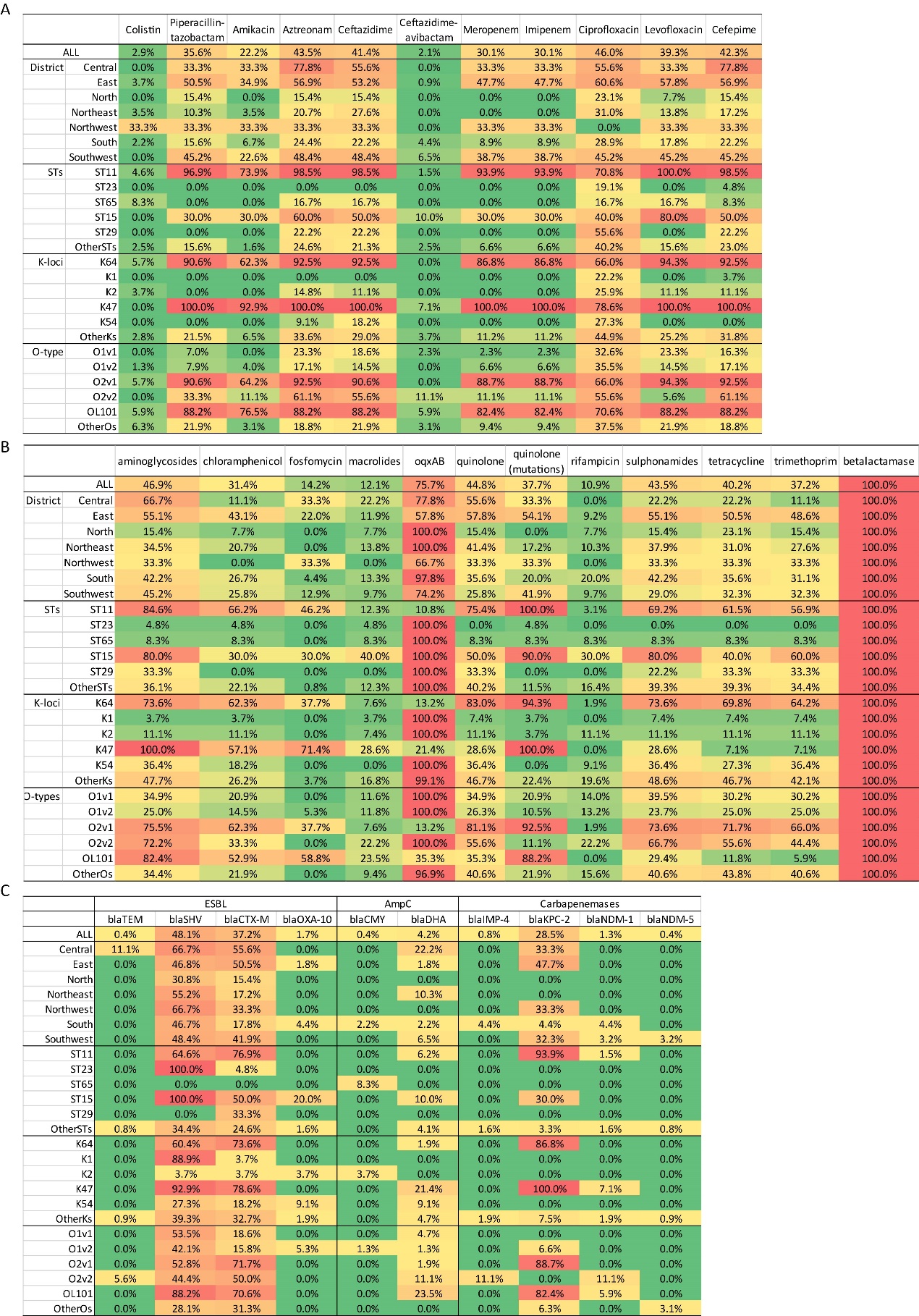


**Figure S4 Antibiotic resistance information of BSI *K. pneumoniae* isolates.** (A) Distribution of antibiotic resistance rate. (B) Distribution of antimicrobial classes for which acquired resistance genes were detected. (C) Distribution of beta-lactamase genes (*bla*_TEM-1_*, bla*_TEM-2_*, bla*_SHV-1_*, bla*_SHV-11_*, bla*_SHV-25_*,* and *bla*_SHV-61_ were excluded).

**Figure S5**


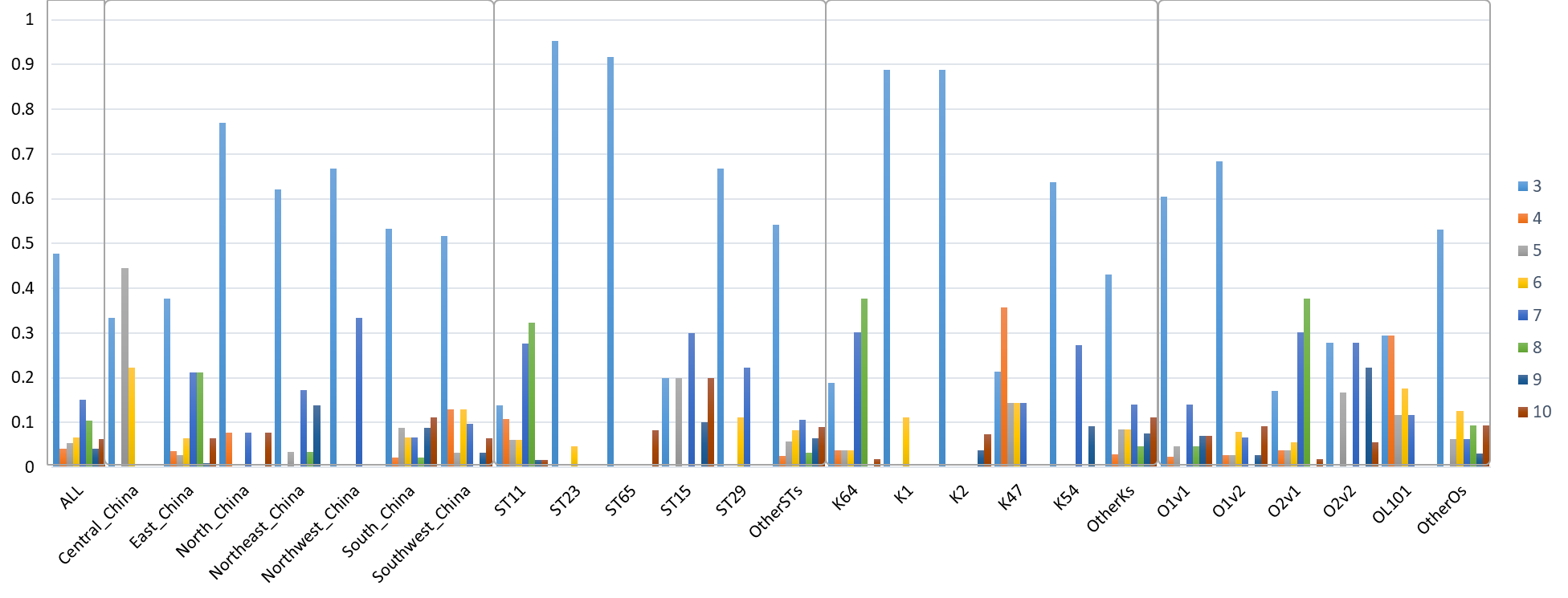


**Figure S5 The number of antimicrobial classes for which acquired resistance genes were detected among different regions, STs, K-loci, and O-type isolates.**

**Figure S6**


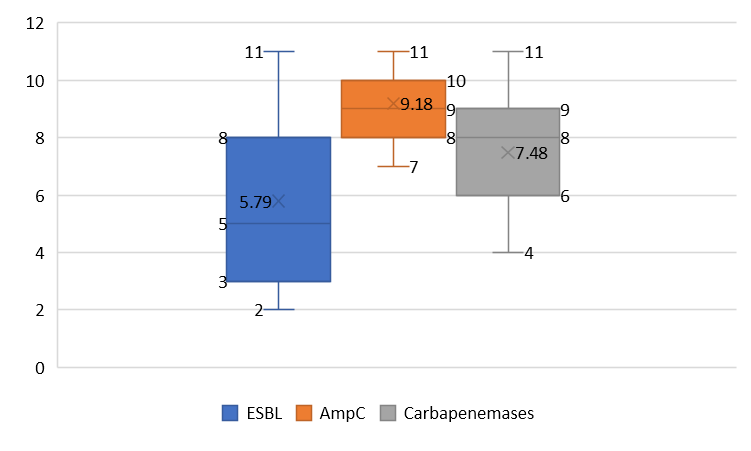


**Figure S6 The number of other antimicrobial classes except for beta-lactamase among the isolates containing ESBL, AmpC, and Carbapenemases genes.**

**Figure S7**


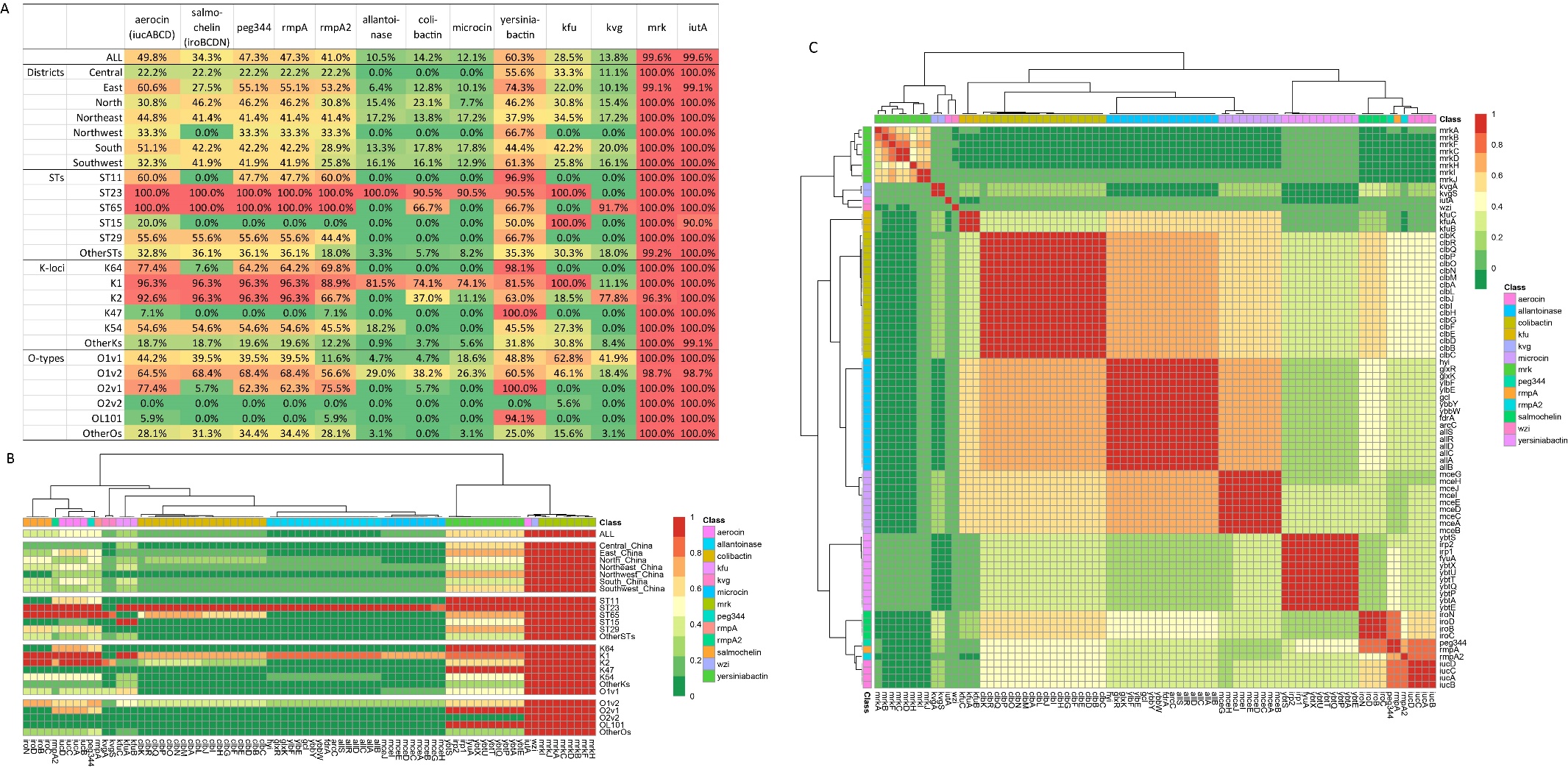


**Figure S7 Distribution of virulence genes of BSI *K. pneumoniae* isolates.** (A) Distribution of virulence gene classes. (B) Distribution of virulence genes. (C) Correlations among virulence genes.


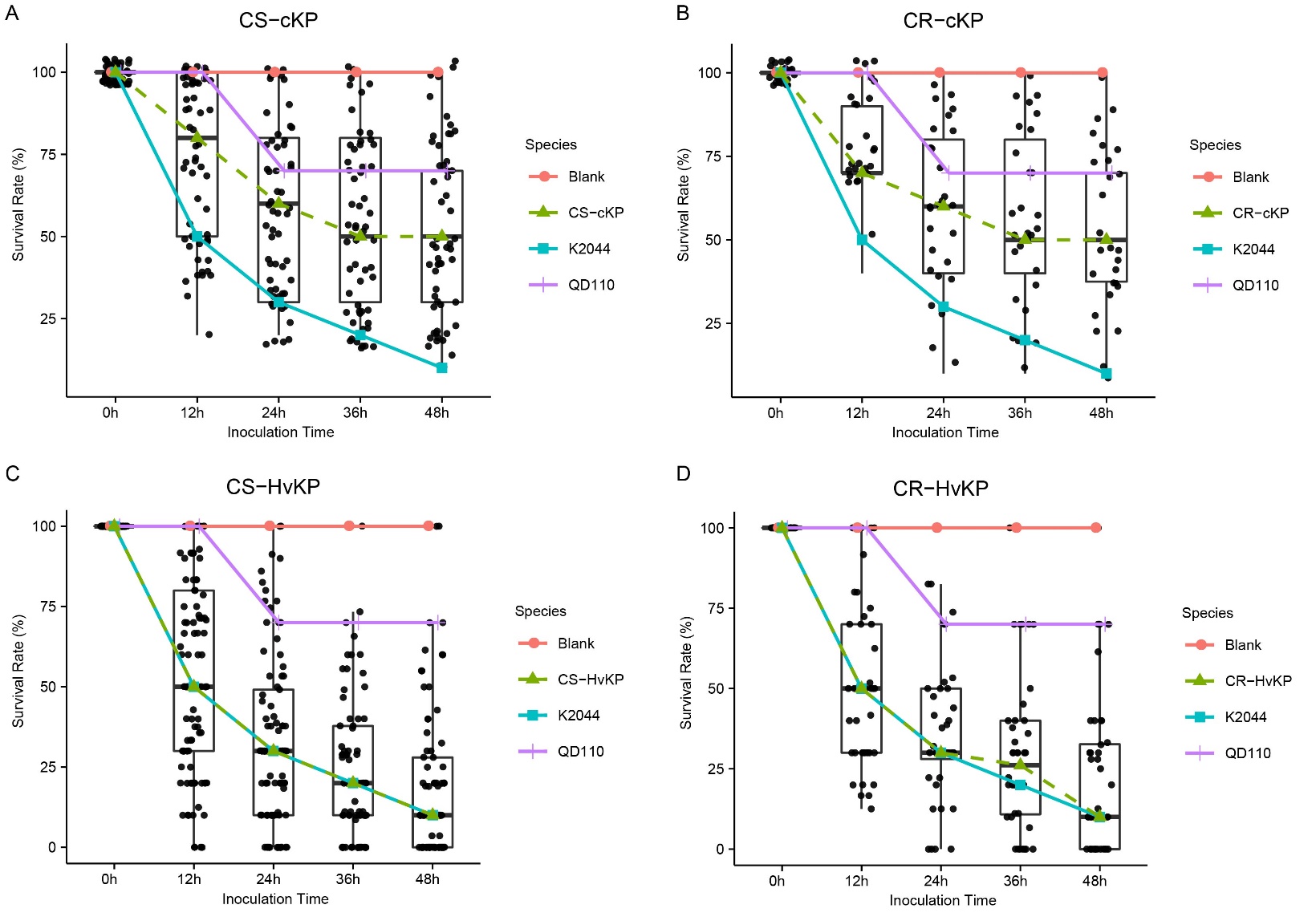
**Figure S8**

**Figure S8 Virulence potential of CS-cKPs, CR-cKPs, CS-HvKPs, and CR-HvKPs in *G. mellonella* infection model.** The effect of 1 × 10^5^ CFU of each strain on survival was assessed in *G. mellonella*. A known hypervirulent *K. pneumoniae* strain NTUH-K2044 was used as the high-virulence control. A classic *K. pneumoniae* strain QD110 identified in this study was added as a low-virulence control. A blank control was also used. All experiments were performed in triplicate.

**Figure S9**


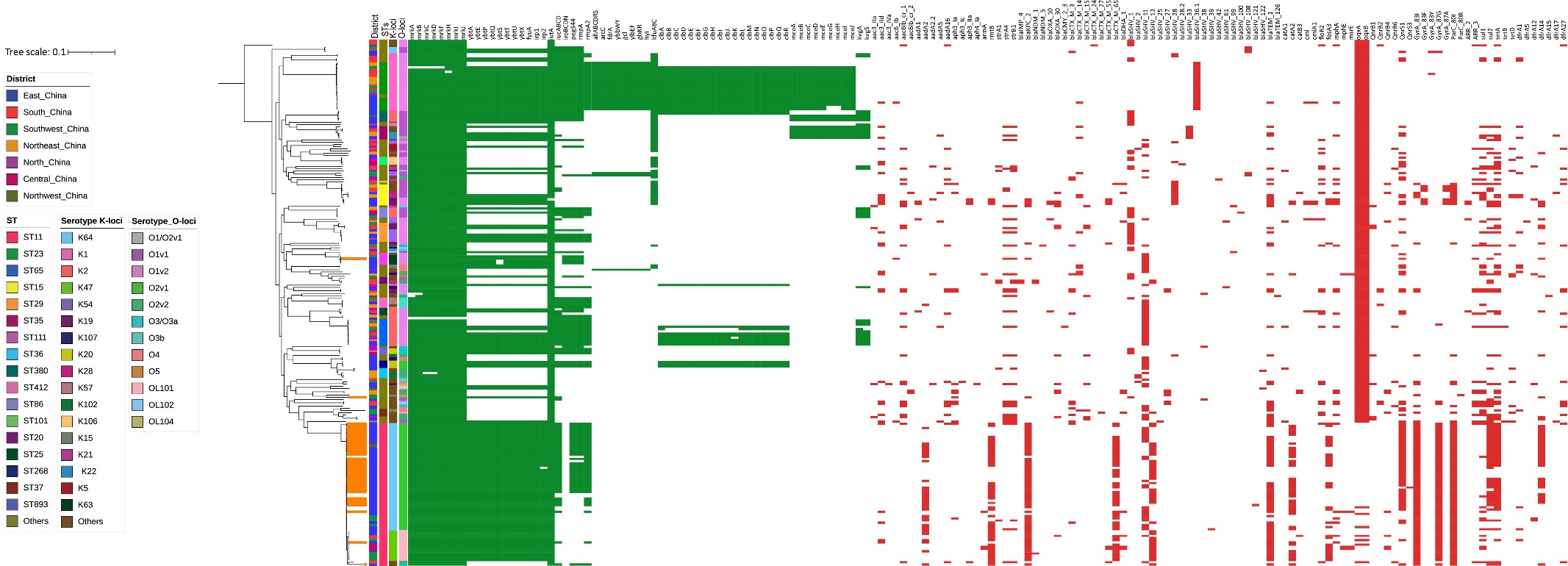


**Figure S9 Evolutionary and distribution of virulence genes and antibiotic resistance genes.** Evolutionary relationships, virulence genes, and antibiotic resistance genes are shown from left to right, respectively. The strains not only contained carbapenemases genes but also hypervirulence genes are highlighted in orange.


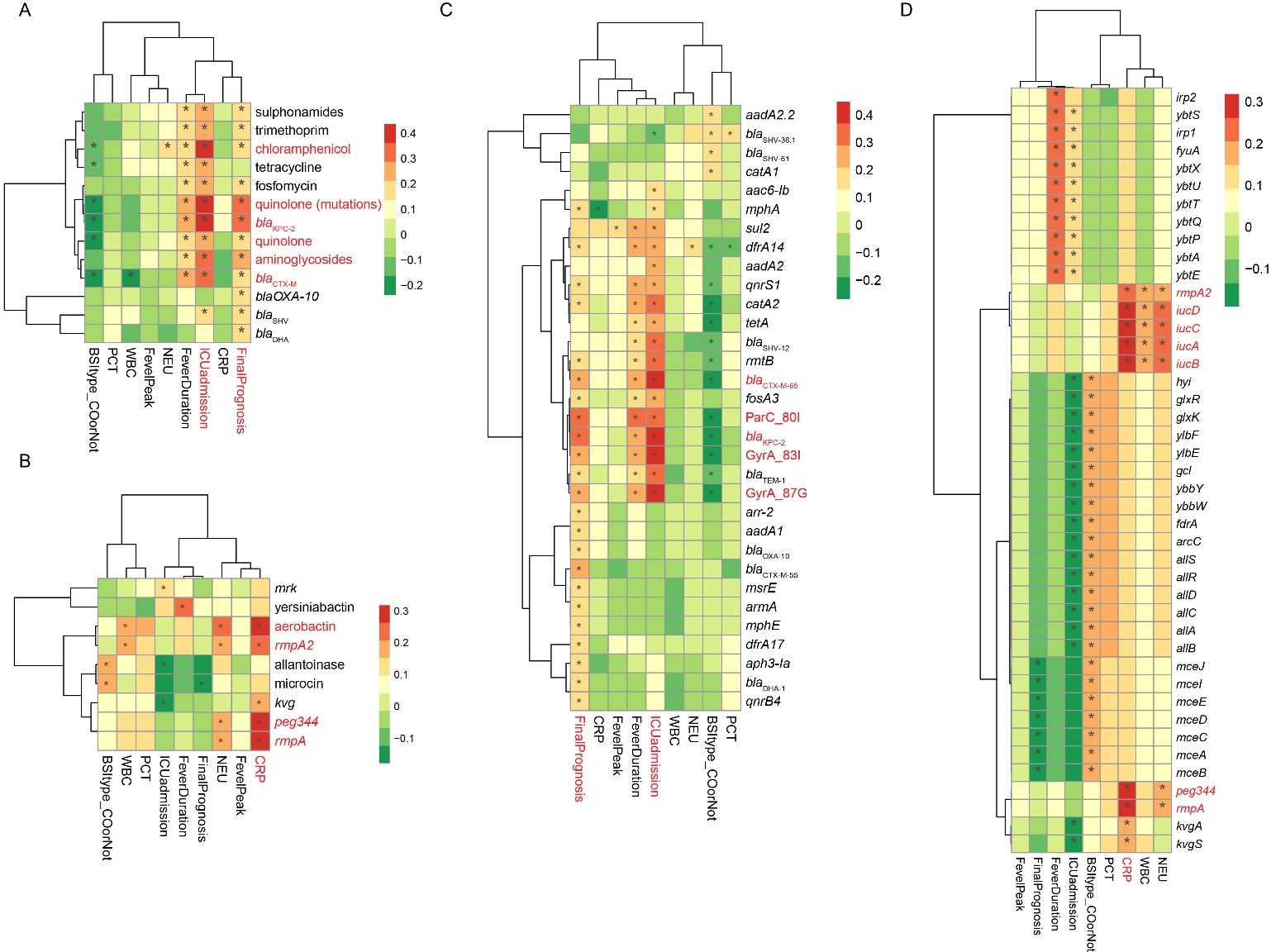
**Figure S10**

**Figure S10 Spearman correlation between genotypes and clinical data in patients with BSI *K. pneumoniae*.** (A) Correlation between antimicrobial resistance gene classes and clinical data in patients with BSI-Kpns. (B) Correlation between virulence gene classes and clinical data in patients with BSI-Kpns. (C) Correlation between antimicrobial resistance genes and clinical data in patients with BSI-Kpns. (D) Correlation between virulence genes and clinical data in patients with BSI-Kpns. Genotype entries that were not significantly associated with any clinical index were excluded from the heatmaps. Cell colours in the main heatmaps correspond to the value of correlation coefficients. Asterisk shows a significant correlation (P < 0.05).

**Supplementary references**

1. Bassetti M, Peghin M, Pecori D. The management of multidrug-resistant Enterobacteriaceae. *Curr Opin Infect Dis*. 2016; 29: 583-94.

2. CLSI. Performance standards for antimicrobial susceptibility testing; Thirtieth informational supplement. CLSI document M100-S30 Clinical and Laboratory Standards Institute: Wayne, PA 2020.

3. Russo TA, Olson R, Fang CT, et al.Identification of biomarkers for differentiation of hypervirulent *Klebsiella pneumoniae* from classical *K. pneumoniae*. *J Clin Microbiol*. 2018; 56: e00776-18.

4. Quan J, Li X, Chen Y, et al. Prevalence of mcr-1 in *Escherichia coli* and *Klebsiella pneumoniae* recovered from bloodstream infections in China: a multicentre longitudinal study. *Lancet Infect Dis*. 2017; 17: 400-410.

5. Gu D, Dong N, Zheng Z, et al. A fatal outbreak of ST11 carbapenem-resistant hypervirulent *Klebsiella pneumoniae* in a Chinese hospital: a molecular epidemiological study. *Lancet Infect Dis*. 2018; 18: 37-46.

6. Yang Q, Jia X, Zhou M, et al. Emergence of ST11-K47 and ST11-K64 hypervirulent carbapenem-resistant *Klebsiella pneumoniae* in bacterial liver abscesses from China: a molecular, biological, and epidemiological study. *Emerg Microbes Infect*. 2020; 9: 320-331.

7. Bankevich A, Nurk S, Antipov D, et al. SPAdes: a new genome assembly algorithm and its applications to single-cell sequencing. *J Comput Biol*. 2012; 19: 455-477.

8. English AC, Richards S, Han Y, et al. Mind the gap: upgrading genomes with Pacific Biosciences RS long-read sequencing technology. *PLoS One*. 2012; 7: e47768.

9. Walker BJ, Abeel T, Shea T, et al. Pilon: an integrated tool for comprehensive microbial variant detection and genome assembly improvement. *PLoS One*. 2014; 9: e112963.

10. Seemann T. Prokka: rapid prokaryotic genome annotation. *Bioinformatics*. 2014; 30: 2068-2069.

11. Inouye M, Dashnow H, Raven LA, et al. SRST2: Rapid genomic surveillance for public health and hospital microbiology labs. *Genome Med*. 2014; 6: 90.

12. Wyres KL, Wick RR, Gorrie C, et al. Identification of Klebsiella capsule synthesis loci from whole genome data. *Microb Genom*. 2016; 2: e000102.

13. Li H, Handsaker B, Wysoker A, et al. The Sequence Alignment/Map format and SAMtools. *Bioinformatics*. 2009; 25: 2078-2079.

14. Koboldt DC, Chen K, Wylie T, et al. VarScan: variant detection in massively parallel sequencing of individual and pooled samples. *Bioinformatics*. 2009; 25: 2283-2285.

15. Stamatakis A. RAxML version 8: a tool for phylogenetic analysis and post-analysis of large phylogenies. *Bioinformatics*. 2014; 30: 1312-1313.

16. Lam MMC, Wick RR, Watts SC, et al. A genomic surveillance framework and genotyping tool for *Klebsiella pneumoniae* and its related species complex. *Nat Commun.* 2021; 12: 4188.
